# Views of Spanish primary care professionals on respiratory tract infection testing: a qualitative study

**DOI:** 10.1136/bmjopen-2025-102476

**Published:** 2025-09-08

**Authors:** Ana Moragas, Josep M Cots, Carl Llor

**Affiliations:** 1 Primary Healthcare Centre Jaume I, Universitat Rovira i Virgili, Tarragona, Catalunya, Spain; 2 La Marina Health Centre, University of Barcelona, Barcelona, Catalunya, Spain; 3 IDIAP Jordi Gol, Barcelona, Catalonia, Spain; 4 CIBER Enfermedades Infecciosas, Instituto de Salud Carlos III, Madrid, Spain; 5 Research Unit for General Practice, Department of Public Health, University of Southern Denmark, Odense, Region Syddanmark, Denmark

**Keywords:** Prescriptions, Diagnostic microbiology, Pulmonary Disease, Chronic Obstructive, Molecular diagnostics, Primary Health Care

## Abstract

**Abstract:**

**Background:**

Respiratory tract infection tests are increasingly available in primary care and are expected to expand in the future. However, there is limited understanding of clinicians’ views on their benefits and how to effectively integrate them into daily clinical practice.

**Objectives:**

The aim of this study was to explore healthcare professionals’ views on using respiratory tract infection tests to safely reduce unnecessary antibiotic prescriptions for respiratory tract infections in primary care based on their experience in routine practice.

**Design:**

A qualitative study design was employed. Two focus group discussions were conducted.

**Setting and participants:**

These focus group discussions were conducted in February 2025, involving 18 Spanish primary care professionals, both experts and non-experts in rational antibiotic use and antimicrobial resistance. Data were audio-recorded or video-recorded, transcribed and analysed thematically.

**Results:**

Participants agreed that respiratory tract infection tests help optimise antibiotic prescriptions, reduce uncertainty, ensure the appropriate consumption of resources and guide treatment based on aetiology. While most professionals view microbiological tests and C-reactive protein testing as complementary, non-experts preferred microbiological tests. Professionals considered that patients value knowing the aetiology over the prognosis, supporting shared decision-making and addressing patients’ demands more effectively. Concerns remain about using these tests as stand-alone tools and the medicalisation of self-limiting conditions. Other barriers mentioned included the high cost and time-consuming nature, the need for better professional training and the challenge of managing the increased workload associated with their use.

**Conclusions:**

This study highlights how clinicians perceive respiratory tract infection tests to aid prescribing decisions amid uncertainty. Both positive and negative views were reported. Participants agreed that these tests optimise antibiotic prescriptions and guide treatment, but there are still important barriers to their implementation.

**STRENGTHS AND LIMITATIONS OF THIS STUDY**

- Although our sample included both expert and non-expert healthcare professionals, primary care doctors who regularly perform molecular testing were under-represented due to the limited use of point-of-care tests for respiratory tract infections.
- The comparatively limited experience of the non-expert group may have constrained the depth of their responses and might have contributed to the uncertainties or hesitations observed.
- Clinicians believe patients prefer tests that identify the cause of an infection over those indicating prognosis, highlighting the need for patient-focused research to clarify their true preferences.
- Focus group discussions continued until data saturation was reached, with two experienced qualitative researchers ensuring rigorous analysis and diverse perspectives.
- While focusing on primary care physicians, we also included nurses, out-of-hours doctors and trainees to broaden the scope of interviewees.

## Introduction

 The judicious use of antibiotics preserves millions of lives and safeguards numerous individuals from infectious complications annually. However, their continued viability is threatened by rising rates of antimicrobial resistance, which was responsible for an estimated 1.27 million deaths worldwide in 2019.^1^ Excessive and inappropriate antimicrobial use significantly contributes to this crisis as research indicates a strong correlation between the consumption of antibiotics and the selection of resistant bacteria, demonstrated at both the community and individual levels.^2 3^ Primary care is responsible for about 80% of antibiotic prescriptions, and respiratory tract infections account for more than half of all antibiotic prescriptions globally, despite most of these infections being self-limiting.^4^ In April 2025, the WHO expanded its primary healthcare framework for addressing antimicrobial resistance. Key priorities include integrating antimicrobial resistance into national health policies and primary care systems, implementing antimicrobial stewardship programmes and conducting community-based and school-based antimicrobial resistance education campaigns. Additionally, a new community platform was launched to facilitate the sharing of best practices.^5^

Antibiotic use exhibits significant variability across both countries and within practices in the same region. The latest data on antibiotic consumption in Europe highlight that overall antibiotic consumption is now slightly higher than it was in the year before the COVID-19 outbreak in 2019.^6^ Although some countries have access to rapid diagnostic tools, such as rapid antigen detection tests for streptococcal pharyngitis and C-reactive protein (CRP) testing, their availability is inconsistent and its use is not routinely implemented in primary care, except in Scandinavian and some other Nordic countries.^7^

Various strategies have been proposed to reduce unnecessary antibiotic use, with some proving more effective than others. The most successful approaches include enhancing communication skills during consultations to better address patient concerns, providing patient leaflets to clarify when antibiotics are or are not needed, implementing delayed antibiotic prescriptions and using point-of-care tests (POCTs) in cases of uncertainty.^8^ Diagnostic uncertainty and the fear of complications are significant reasons why doctors may hesitate to refrain from prescribing antibiotics when uncertain.^9^ Rapid microbiological POCTs have emerged as valuable tools for improving diagnostic accuracy, capable of detecting viruses and bacteria from respiratory tract samples in as little as 15 min using nucleic acid or antigen-based methods. These tests fall into two main categories: molecular tests, which detect nucleic acids, and antigen tests, which identify specific antigens.^10^

While multiplex POCTs have shown promising results in reducing hospital admission times and length of antibiotic courses in secondary care,^11^ more research is needed to explore primary care clinicians’ perspectives on the integration of these tests into primary care and to gain greater insight into how respiratory tract infection tests can influence clinical decision-making and patient management.

## Methods

### Study design

A qualitative study design was employed, using semistructured topic guide conducted within two focus group discussions (FGDs) involving primary care healthcare professionals. The semistructured topic guide format provided a consistent framework across both sessions while allowing for open-ended responses and facilitating in-depth discussions among participants. The topic guide was developed by authors AM and CL, based on a review of the relevant literature, and served as a thematic anchor to initiate and guide the conversations in both FGDs.

The FGDs were video-recorded with the oral consent of participants in the online focus group and written consent from those participating in the in-person group who were audio-recorded. The recordings were transcribed by the two lead authors using computer-assisted transcription software (Google Docs Voice Typing).

### Recruitment and sampling

Two FGDs were conducted: one with experts (E) in infectious diseases, rational use of antibiotics and antimicrobial resistance, and another with non-experts (NE). Participants were selected through a convenience sampling strategy. The research team consists of AM, a general practitioner at a primary care centre, associate professor at Rovira i Virgili University and researcher at the Jordi Gol Institute for Research, and CL, a researcher at the Jordi Gol Institute, affiliated with CIBER and the University of Southern Denmark. Both have experience in quantitative and qualitative research on infections in primary care and have previously collaborated with some participants through the Spanish Society of Family Medicine research group.

Due to the geographical dispersion of the expert participants across Spain, a virtual FGD was held via Microsoft Teams. In contrast, the NE group was able to meet in person. The E group included a range of healthcare professionals such as general practitioners, nurses, general practitioners working in out-of-hours primary care services and clinicians from both rural and urban settings. Members of the Infectious Diseases Study Group of the Spanish Society of Family Medicine were specifically chosen for their recognised expertise in the use of rapid diagnostic tests. Some participants had previously collaborated with the research team in earlier studies. In addition to these members, a few doctors not affiliated with the study group but recognised for their expertise in rapid testing, as well as a family medicine trainee with similar expertise, were also included. The NE group comprised family medicine trainees, family nurse trainees and their clinical tutors (table 1). Most E group participants were already familiar with the researchers through previous collaborative work within the Spanish Society of Family Medicine. This group included nine professionals, one moderator (AM) and one observer (CL). The NE group also consisted of nine participants and was moderated by AM without an observer. Participants in the NE group were acquainted with the researcher, as they worked in nearby primary care centres. Both FGDs were facilitated by the same researcher (AM).

**Table 1:** Participant characteristics

Participant	Gender	Years working	Rural/urban centre	Workplace	Profession	Experience in performing nucleic acid tests	Experience in performing antigen tests
Expert group							
E1	Male	Retired	Urban	Primary care	Family medicine	>3 years	>5 years
E2	Female	>10 years	Urban	Emergency department	Family medicine	<3 years	>5 years
E3	Male	>20 years	Urban	Emergency department	Family medicine	>3 years	>5 years
E4	Female	>20 years	Urban	Primary care	Nurse	0 years	>5 years
E5	Female	3 years	Urban	Emergency department	Family medicine trainee	0 years	3 years
E6	Male	>20 years	Urban	Primary care	Family medicine	>3 years	>5 years
E7	Male	>20 years	Rural	Primary care and emergency department	Family medicine	>3 years	>5 years
E8	Male	>20 years	Rural	Primary care and emergency department	Family medicine	>3 years	>5 years
E9	Female	>20 years	Urban	Primary care	Family medicine	0 years	>5 years
Observer	Male	>20 years	Urban	Primary care	Family medicine	0 years	>5 years
Non-expert group							
NE1	Female	>20 years	Urban	Primary care and emergency department	Family medicine	0 years	>5 years
NE2	Female	>30 years	Urban	Primary care	Nurse	0 years	>5 years
NE3	Female	2 years	Urban	Primary care and emergency department	Nurse trainee	0 years	2 years
NE4	Female	2 years	Urban	Primary care and emergency department	Nurse trainee	0 years	2 years
NE5	Female	5 years	Urban	Primary care and emergency department	Nurse trainee	0 years	>5 years
NE6	Female	3 years	Urban	Primary care and emergency department	Family medicine trainee	0 years	3 years
NE7	Female	3 years	Urban	Primary care and emergency department	Family medicine trainee	0 years	3 years
NE8	Male	1 year	Urban	Primary care and emergency department	Family medicine trainee	0 years	1 year
NE9	Male	4 years	Urban	Primary care and emergency department	Family medicine trainee	0 years	4 years

E, expert; NE, non-expert.

The expert FGD lasted approximately 75 min, while the non-expert session lasted 45 min. E group participants were recruited via email: an initial message outlining the purpose of the session was sent, followed by a reminder with the meeting details. All invitees responded, with only one unable to attend due to scheduling conflicts. The NE group was recruited during a Trainee Day event at a primary healthcare centre, where researcher AM explained the study’s purpose. All approached individuals agreed to participate. The E group provided oral consent, which was recorded during the session, while the NE group gave written consent. The expert session was conducted online and both audio and video were recorded. The NE session was held in person, with only an audio recording made. Researchers took notes during both sessions and shared them for discussion afterward. Data saturation was assessed collaboratively following the FGDs. Transcripts, the coding tree and feedback were shared with a subset of participants. Due to the difficulty in contacting all study members because of their dispersion, we decided to engage only a selected group to facilitate the feedback process. The topic guide is detailed in box 1.

Box 1 **Topic guide on respiratory tract infection rapid testing**

1. What types of rapid diagnostic tests are used in your health centres?
2. What do you know about rapid diagnostic tests?
  - In which diseases do you think point-of-care tests (POCTs) could be helpful?
  - Have you ever used POCTs for community-acquired infections? Have you ever used POCTs for community-acquired infections?
  - What do you know about molecular POCTs? In which conditions do you think they might be useful?
  - What are the differences between conventional and molecular POCTs?
  - What are the pros and cons of molecular tests?
  - Where do you think POCTs (both molecular and general) could be most useful: primary care, home visits, nursing homes, emergency services…?
3. Do you think POCTs can support antibiotic prescribing for community-acquired infections?
  - What benefits could POCTs offer?
  - If there were a POCT capable of distinguishing between viral and bacterial infections, what impact would it have on your clinical practice?
  - Would you prefer a test that informs you about the aetiology of the infection or one that informs you about the prognosis (eg, severity) of the infection?
4. What do you expect from a POCT?
  - What do you think is important and what is not?
  - Sensitivity? Specificity? Accuracy?
  - Do you think it would be easy to implement in our primary care consultations?
  - Is it cost-effective?
  - Would you prefer a quantitative or qualitative test?
  - What would be the maximum acceptable time to obtain results in daily primary care practice?
5. What barriers or challenges do you foresee for introducing POCTs in your health centre?
  - How and where would you implement them?
  - Who should be responsible for carrying out the test?
  - Some centres have POCTs available but do not use them—why do you think that happens?
  - What problems might arise, and how would you solve them?
6. What impact do you think POCTs might have on patients and the doctor–patient relationship?
  - Do you think POCTs reduce uncertainty in diagnosis and decision-making?
  - Do they improve communication with patients?
  - Do they improve patient satisfaction?
  - Do you think there is a difference in impact between tests that provide aetiological information and those that provide prognostic information?
7. What information would you need about a POCT for infections to feel confident introducing it in your primary care setting?
  - Test cost?
  - Sensitivity?
  - Specificity?
  - Role of clinical guidelines?
  - Who would you prefer to communicate this information to you?

### Data collection

Two researchers (AM and CL) collected data using semistructured topic guides. The authors reviewed the relevant literature to develop the topic guide. A pilot test was not conducted. Clinicians were asked about their experiences in managing rapid POCTs. Data were collected in February 2025 and subsequently analysed.

### Analysis

The transcripts were checked for accuracy against the FGD recordings, then anonymised and analysed using thematic analysis. This method was chosen for its flexibility and suitability for exploratory studies, enabling a detailed understanding of healthcare professionals’ perspectives on rapid microbiological testing in primary care. Thematic analysis is particularly appropriate for focus group data, as it captures both explicit and implicit meanings and provides a transparent, rigorous framework for coding and theme development, enhancing the study’s credibility.

We followed the six iterative steps of thematic analysis: familiarisation with the data, generating initial codes, searching for themes, reviewing themes, defining and naming themes and producing the report.^12^ Two researchers (AM and CL) served as primary coders, applying primarily inductive and semantic coding, supported by NVivo V.12 software. A line-by-line approach was used to systematically code meaningful units related to the research questions, with similar codes grouped into categories. Constant comparison across FGDs enabled the identification of patterns, contrasts and deviant cases. The E group FGD was double-coded to enhance analytical rigour, while the NE group was coded solely by the moderator due to feasibility constraints. Reflexive awareness was maintained throughout, with reflective memos used to track the influence of researchers’ backgrounds and potential biases on data collection and analysis.

### Patient and public involvement

No patients were recruited for the study, and none were involved in its design or in the interpretation of the results.

## Results

A total of 18 primary care professionals participated in the FGDs. 11 females and 9 males, most with more than 20 years of working experience (table 1). All participants expressed both positive and negative views about the use of POCTs in primary care. A total of four themes emerged (table 2). The NE group demonstrated comparatively less experience, which may have limited their ability to respond comprehensively to certain questions. This difference in experience could explain some of the uncertainties or hesitations observed in their responses.

**Table 2:** Positive and negative views about point-of-care tests for patients with respiratory tract infections

Themes	Positive views	Negative views
Practicalities of rapid microbiological tests	The validity of molecular point-of-care tests is higher than that of antigenic tests.	The availability of tests is irregular; molecular tests are only available in some places. It is important to understand how the test works in order to obtain reliable results. Molecular tests are more expensive than antigenic point-of-care tests.
Impact on clinical decision making	Tests help professionals optimise antibiotic prescribing. Aetiology helps determine the treatment. Microbiological tests and CRP are considered complementary; however, non-experts preferred microbiological tests. Combining utilisation of rapid tests with other strategies, such as delayed antibiotic prescribing, is more effective. Tests can help ensure the appropriate consumption of resources. Tests are especially useful in older people.	Rapid tests are often used outside their intended purpose, and there is a risk of them being used as stand-alone tests. Rapid tests do not exclude the need to take a proper medical history and perform a correct physical examination.
Impact on doctor–patient relationship	Patients appreciate being tested. Tests reduce uncertainty for doctors and patients. Professionals think that patients prefer to know the aetiology rather than the prognosis. Tests are useful as an educational tool for patients. Tests address patients’ demands more effectively and improve adherence. Tests facilitate shared decision-making.	Fear of medicalisation and the risk of increased reattendance for self-limiting conditions.
Integrating tests into practice	Qualitative tests provide quicker results. Quantitative tests are easier to read.	Molecular tests are expensive. Time-consuming. The need to improve training for professionals. The increased workload associated with their use.

CRP, C-reactive protein.

### Theme 1: practicalities of rapid diagnostic tests

The availability of rapid diagnostic tests for respiratory infections depends greatly on the region. With the development of the antimicrobial stewardship programmes in primary care, most centres now have rapid antigenic diagnostic tests for streptococcal infection. However, the availability of CRP rapid tests is more irregular. Currently, almost all centres have antigenic tests to detect COVID, Influenza A virus and Influenza B virus, although protocols may differ. Molecular tests are available only in very specific locations, primarily in emergency departments and rural areas.

We have Strep A tests and COVID tests, but we don’t have any other tests. In our area, the hospital is close by, and if there’s any doubt, the patient is referred to the hospital (E9).

We have been working with molecular tests since 2022, and we are now conducting a study on the usefulness of molecular diagnostic tests in rural areas, mainly because we believe this is where they could be most efficient (E8).

In the emergency department, we have the Strep A antigen test, we also have Legionella and Streptococcus pneumoniae urine tests, and we have also had a multi-virus test for respiratory infections for the past two months (E3).

Participants emphasised the need to thoroughly understand how these devices work in order to ensure reliable results, as well as the need for training on how to perform the techniques.

You should read the instructions carefully and follow them exactly. Sometimes we rush and don’t wait long enough. For example, there are these antigen tests that cover RSV, flu A, flu B, and they say you have to leave the swab in for at least 30 seconds. It’s uncomfortable for the patient, but it’s necessary for the test to work properly (NE5).

It’s just that, of course, we switched from the COVID test to the Multitest, and it’s different. I think times have changed, and you just do things the way you’re used to, but that’s not right. These are new tests, and you need to review them (NE1).

Regarding the main differences between molecular and antigenic POCTs, participants agreed that molecular devices are more reliable, although they also come with a higher cost, which limits their implementation nowadays. They believe that molecular tests might be implemented in the future, along with the use of artificial intelligence.

Well, they are more reliable, it’s also true that they take longer, but they are more reliable. The main concern is the cost, and this limits the implementation nowadays, but this might change in the future (NE6).

The sensitivity of molecular tests is much higher, I think in that regard, it discriminates better. The cons, obviously, are the high cost of these tests (E8).

In the future, artificial intelligence will have an algorithm that will tell you whether the test is indicated or not. I think this is going to evolve very quickly, and it will really prevent a lot of consultations in the hospital setting (E3).

Several clinicians expressed concerns about interpreting test results, highlighting that positive findings may not always indicate a causative infection. They noted that some detected organisms could be commensals or colonisers, particularly in cases of mixed bacterial–viral respiratory tract infections.

Healthcare professionals required the guidelines established in clinical protocols to be clear and specific in order to make informed decisions about when it is most appropriate to use one technique over another. For this reason, there is a recognised need for more studies that provide solid evidence and concrete recommendations.

### Theme 2: impact on clinical decision-making

Professionals assured that tests help them; on one hand, to understand how they should act and give them an idea of the severity or the causative agent—this could be through CRP or aetiological tests aimed at identifying the pathogen—to determine whether clinicians need to refer the patient to the hospital or if they can be managed in primary care. On the other hand, the tests help clinicians optimise antibiotic prescriptions, aiming to reduce inappropriate overuse of antibiotics.

The key point is that both CRP and microbiological tests help guide patient management. CRP tests, in particular, help distinguish between viral and bacterial infections by indicating the level of inflammation, which can influence decisions like whether to refer a patient to the hospital or manage them in primary care. These tests reduce uncertainty and can either reaffirm the current treatment approach or prompt a change in management, depending on the results. Similarly, microbiological tests provide important information about the cause of an infection, also impacting treatment decisions (E3).

To me, the first consequence, which is much more important for everyone in general, is whether the patient needs an antibiotic or not, because antibiotic prescription is completely modified with the use of rapid diagnostic tests (E6).

Regarding whether clinicians prefer techniques that provide information on aetiology, such as microbiological tests, or those that support prognostic assessment, such as CRP rapid tests, which can also contribute to the diagnostic process, participants were divided in their preferences when deciding on antibiotic treatment. Most considered that both types of rapid tests are complementary.

However, some experts, and mainly non-experts, preferred knowing the aetiology, as identifying the pathogen helps determine the treatment more effectively. However, non-experts valued microbiological tests more, with some arguing that knowing only the prognosis does not guarantee appropriate treatment. Some experts still preferred having prognostic information, although all agreed that the two approaches are complementary. All the clinicians, however, recommend complementing the utilisation of tests with other strategies, such as enhanced communications skills or delayed antibiotic prescribing.

I prefer knowing the aetiology, but I don’t think they are mutually exclusive. With the clinical signs, we can know the prognosis. And knowing the aetiology also gives you the prognosis, right? We have the microbiological tests, and we still use CRP for certain situations in which the aetiology isn’t clear, for example, when tests are negative, and you have a lower respiratory tract infection. In the end, your doubt is whether it’s bacterial or viral and whether you need to give an antibiotic or not (NE1).

It’s also true that if you know the aetiology, you know how to treat it. The prognosis doesn’t matter if you don’t know how to treat it. If you have a microorganism, you know a bit about the prognosis as well—Pseudomonas is not the same as E. coli, for example (NE7).

I think they’re complementary. What really interests me is the aetiology, and based on that, I’ll decide how to treat. However, even if it gives me the aetiology and tells me it’s pneumococcus, I’d still have the same doubts because what I really need to know is how it might evolve. In this case, CRP gives me more information because it shows if there’s a significant inflammatory response. It depends. Tests are important, but communication with patients is key (E6).

I prefer the prognosis. Sometimes I use the delayed antibiotic prescribing or simply ask patients about what they think about antibiotic therapy. I prefer reducing the uncertainty about whether the patient has a severe condition. After that, I will look for the aetiology. CRP, above all, is helpful when you get a very low value to ease your mind (E7).

Another benefit of using POCTs is that they not only help with the proper use of antibiotics but also with the appropriate consumption of resources.

Knowing the aetiology of an infection is key. The tests help us better assess how severe a patient’s condition is and whether they’re at higher risk of needing hospitalisation. Plus, using rapid diagnostic tests helps cut down on resources, consultations, emergency room visits, extra tests, and so on (NE9).

Microbiological tests are also useful during epidemic periods, especially to decide if isolation of the patient is necessary (E7).

Most healthcare professionals thought that rapid tests were suitable for use in a range of patients, especially older people, because infections caused by certain bacteria and viruses can be more serious in this age group and knowing this is useful for closer monitoring. Additionally, there is a tendency among prescribers to use more antibiotics in this age group.

When I visit an old person with a lower respiratory infection and they have difficulty breathing, I ask for a COVID-19, flu, and RSV test; it only takes 15 minutes but this gives me a lot of information, because I know that if they test positive for RSV for example, they could have a worse outcome compared to when everything is negative. It’s important to be cautious with older people. The same goes for the flu; it’s also useful because it helps them take measures to prevent spreading the infection to others (NE7).

It was also discussed that it is important to meet the criteria before conducting the test, and that in many cases, rapid tests are used outside their intended purpose. Experts, in particular, expressed concern that over-relying on these tests could lead to their use as substitutes for, or even before, a clinical examination is done by many healthcare professionals. This issue is already occurring with CRP rapid testing in some Scandinavian countries.^13^

No one would think of performing a test without knowing when to use it, and furthermore, there must be a restrictive protocol in place. That is, it should be used when I’m considering prescribing an antibiotic and have doubts, not when I’m certain I won’t prescribe it (E6).

A clear example is the CRP test. It is often used outside of its intended indications… and it turns out that when a person presents with a fever, the first thing they do is order a CRP. This could be the same situation with these microbiological tests. I’m concerned that, as happens in some places, they could be used even before examining the patient, and this shouldn’t be the case (E7).

### Theme 3: impact of rapid tests on the doctor–patient relationship

All participants agreed that patients appreciate being tested, feel better cared for and that it improves the doctor–patient relationship. Participants claimed that knowing the aetiology of the infection reduces the uncertainty of professionals, as well as reduces the fear of patients, making them feel more assured and more satisfied.

As for the patient, all the tests you perform make them happy; in other words, anything that gives objective knowledge from a test. The more tests you do on the patient, the happier they will be, even if they are unnecessary. We live in a consumer society, and this is just another form of consumption (E7).

I think the patient loves it and feels very assured because it’s like you are taking care of them properly, and when it comes down to satisfaction, both professionals and patients are pleased (E8).

It gives more confidence to the professional, and the patient has more confidence in you. When I need it, my doctor or nurse performs the tests I need (NE2).

The professionals also agreed that they think patients prefer to know the aetiology of the pathogen, as it provides them with a greater sense of security, rather than knowing the prognosis.

If you go to a health centre with a fever and you’re told, ‘You have influenza A, and you should take paracetamol’, the patient will feel more convinced. In general, if you don’t give an antibiotic, you also have more arguments, and the patient feels more at ease (E6).

Likewise, patients feel more at ease with the aetiology because they always want to know what they have, a specific diagnosis. It gives you security. You also feel more confident telling them, ‘This is a virus. I’m sure, and this test confirms it’ (NE1).

It was also mentioned that the use of these tests facilitates shared decision-making and improves treatment adherence. Some participants also stated that one of the advantages of these rapid tests is that healthcare professionals can more effectively convey to patients who ask for or expect an antibiotic that one is not needed if the infection is caused by a virus, compared with using biological markers.

I believe it does have an impact, especially on adherence for those who test positive. I see that with the Strep A, patients are more convinced to take the antibiotic as requested if the result is positive, and they are more assured if the result is negative. I think it helps reduce the inappropriate use of antibiotics and prevents self-medication, because if they leave the consultation with a negative result and their doctor tells them they don’t need antibiotics, well, they’ve gained trust in the doctor, and you can even involve them in the decision-making process. It’s a tool to reduce dissatisfaction (E7).

It gives them peace of mind, and it gives us peace of mind too. If the test is done, they leave more convinced. There are still many patients who come to the consultation either for sick leave or because they want an antibiotic. If you tell them, ‘Your infection is caused by a virus and is self-limiting’, you can address their demand more effectively (NE2).

Professionals also agreed that it is useful for educating patients and teaching them how to act in the future.

The use of these tests increases the trust and relationship between the patient and the professional, and at the same time, they can be used as a tool for health education for the patient, for the future, to know when and how to use healthcare resources properly (E9).

Apart from the positive aspects of using rapid tests in the clinician–patient relationship, the concern of medicalisation and the risk of increased reattendance for similar conditions emerged as drawbacks. While the use of these tests can be helpful in some situations, their widespread use should be avoided for self-limiting conditions.

I’m also not sure if starting to use these tests routinely could lead to the medicalisation of self-limiting conditions, resulting in more appointments in the future as patients want to know what is causing their infection (E6).

### Theme 4: integrating the rapid tests into routine practice

Participants are divided on how these rapid tests should provide results, but most prefer quantitative tests that offer quick results, are easier to interpret and are inexpensive.

I think molecular microbiological tests are the future, as long as they’re easy to obtain, inexpensive, and affordable for the administration to purchase. Personally, I’d prefer them to be quantitative, meaning a numerical value, not just a positive or negative result (E7).

In primary care, qualitative is better because it’s faster, it’s either yes or no, and it guides you (NE5).

Professionals considered that tests should provide results as quickly as possible and should also be cheaper.

Even though the current testing device has improved, the diagnosis takes between 5 and 10 minutes. Okay, so when they’re positive, they show up in 5 minutes, but for them to be negative and for you to be certain they are negative, you must wait between 10 and 15 minutes (E8).

It was also discussed that there is misuse*—*mainly overuse*—*of POCTs and the need to improve training for professionals:

It’s necessary to better define when the test should be performed and the exact criteria for using it correctly. This impacts the economy, as it results in a misuse of resources. When we were first provided with the CRP, there was practically no use in the first few months. The use increased progressively, and now it has skyrocketed. We’ve flipped the script; now, when a patient with a fever visits a continuous care point, the professional automatically performs a CRP. This should not happen with the rapid microbiological tests (E9).

All the emergency departments have CRP, and it’s true that it’s useful if used correctly, but I’m not sure if in some situations its use is correct. However, it’s true that emergency departments do not tend to use CRP in capillary blood, I don’t know why, but practically no one uses it when indicated, while in health centres, it’s the opposite, it’s overused. However, we use microbiological tests more often, and perhaps we should have clear indications of when to use them and when not to (E5).

I’m concerned about the overuse of rapid microbiological tests. There needs to be more emphasis on training, both for nursing and doctors, especially regarding the usage protocols (E1).

One topic discussed was the increased workload as a barrier to implementing POCTs and the need for each centre to find solutions to alleviate this burden, particularly in nurse offices.

I think the biggest issue is the fear of the workload—saying, now I must do the test, who’s going to do it, and until I get the result, I’m just complicating the situation. When the easiest thing is to just give the patient an antibiotic. In my opinion, this should be centralized in a single nurse’s office and clearly outlined in the centre’s protocol (E4).

I believe the decision shouldn’t be exclusively up to the doctor, but rather the healthcare professional attending to the patient. Above all, we need to adapt to the internal processes of the healthcare centres. Another potential barrier could be the usual complaint—the increased workload we face every day (E6).

## Discussion

### Summary of the main findings

Clinicians value rapid tests for improving clinical decision-making and patient satisfaction. Professionals believe that patients are generally more familiar with understanding aetiology than prognosis. Integrating these tests into practice could enhance communication between clinicians and patients, helping to determine whether antibiotics are necessary. Clinicians find these tests particularly valuable when patients ask for or expect antibiotics, and especially in older adults. However, barriers such as high costs, time consumption and the potential for over-reliance hinder their widespread adoption. While most professionals view microbiological tests and CRP testing as complementary, non-experts tend to prefer rapid microbiological tests.

However, most agreed to combine the use of these tests with delayed antibiotic prescribing or enhancing communication skills. Despite supporting shared decision-making, concerns include the risk of overmedicalising self-limiting conditions, insufficient training and increased workload associated with the use of these tests.

### Comparison with existing research findings

Our research builds on previous studies of CRP POCT, suggesting that rapid testing can increase clinicians’ confidence in refraining from prescribing antibiotics, thereby reducing concerns about potential negative clinical outcomes when antibiotics are withheld.^1214^,^16^ This boost in the clinician’s self-confidence, particularly when test results point to a viral cause, is especially beneficial. Non-experts showed a greater interest in rapid microbiological testing, while the more experienced individuals relied not only on microbiological tests but also on CRP testing, as the latter provides a better prognosis for respiratory tract infections. In this context, the use of these rapid microbiological tests could be particularly advantageous for less experienced clinicians.

During the course of writing this paper, a similar recent study conducted in the UK was published.^17^ This study also highlighted an over-reliance on microbiological tests among less experienced clinicians and discussed both the potential benefits and challenges of integrating rapid microbiological POCTs into routine clinical practice. Clinicians in that study generally expressed favourable views towards the use of rapid microbiological testing.

Unlike the British study, which is a clinical trial focused on molecular POCTs, our study is set within real-world clinical practice and includes all types of available tests.^17^ Our study also emphasises that the use of rapid microbiological tests can play a valuable role in managing patient expectations, particularly by providing greater clarity about the cause of infection. However, this benefit may come with a risk. If clinicians begin to over-rely on these tests, using them prior to conducting a thorough clinical examination or treating them as standalone diagnostic tools, it could potentially undermine essential clinical skills and judgement, as observed with CRP rapid testing in some Scandinavian countries.^13^ This could lead to the overutilisation of rapid tests, surpassing the clinical recommendations in guidelines. In our study, clinicians recognised the value of rapid tests in addressing patient expectations regarding antibiotics and improving overall patient satisfaction. These results are consistent with prior research showing that both CRP and rapid microbiological POCTs support prescribing decisions and provide reassurance by identifying the underlying cause of the infection.^18^,^20^ Most participants in our qualitative study believed that patients are more accustomed to the viral/bacterial differentiation than to the mild/severe classification of a respiratory tract infection. As a result, patients would prefer to know the aetiology of the infection. Consequently, this serves as a valuable communication tool that enhances the doctor–patient relationship, leading to better adherence to the doctor’s recommendations. The fact that using these tests can reassure patients requesting antibiotics, or even when healthcare professionals perceive that patients want antibiotics, aligns with other studies conducted with different rapid tests, such as CRP testing.^21^

The main barriers to implementing rapid tests are the high cost and the still considerable time required, especially with molecular tests, as well as how to integrate these tests into routine practice, as also mentioned in the recent paper referred to previously.^17^ One concern raised in our study was the potential high workload that the use of these tests might create in the future. The fear of medicalisation and patient reattendance for similar conditions in the future is also a barrier mentioned by healthcare professionals; an issue also observed in qualitative studies using CRP.^22^ This highlights the need for a clear understanding of when these tests should and should not be used, along with well-defined indications and guidelines.

## Strengths and limitations

This qualitative study aimed to explore the perspectives of primary care professionals on rapid tests for respiratory tract infections in a specific region of Southern Europe, where antibiotic consumption is notably high. It is uncertain whether different viewpoints might emerge in areas with lower antibiotic prescription rates. However, the study included both experts who advocate for the rational use of antibiotics and are particularly concerned about the antimicrobial resistance threat and those aware of the issues related to antimicrobial resistance, as well as non-experts. The focus was specifically on primary care clinicians working in publicly funded clinics, as these settings are where the majority of antibiotic prescriptions occur. Participants included experienced doctors managing respiratory infections in primary care settings, including out-of-hours services, as well as young family medicine trainees. It is acknowledged that the NE group possessed comparatively less experience, which may have limited the comprehensiveness of their responses to certain questions. This difference in experience may explain some of the uncertainties or hesitations observed during their contributions. It should also be acknowledged that the majority of participants from both the E and NE groups had experience using antigen tests, whereas their expertise with molecular tests is comparatively limited.

We did not limit our scope to only clinicians from a range of clinics to reflect the breadth of antibiotic prescribing practice in this region. Nurses and nursing trainees were also included, as they are increasingly responsible for managing these common infections in many European countries. We did not recruit patients for this qualitative study. The fact that clinicians believed patients would prefer to know the aetiology rather than the prognosis of the infection could have been explored further with the inclusion of patients. However, we are planning to conduct a study with patients to explore their views on the use of these tests in primary care.

The study has several notable strengths. The convenience sample was well-balanced, featuring both infectious disease experts and those less specialised in the field. Despite including a balanced number of expert and non-expert healthcare professionals, the number of primary care doctors working in primary care consultations who routinely performed molecular testing was underrepresented in our sample, as these POCTs for respiratory tract infections are not currently used routinely in this setting. FGDs continued until enough data were gathered to develop well-supported themes. The involvement of two experienced qualitative researchers in data collection, analysis and interpretation ensured that a range of perspectives and insights were considered, providing a deeper and more comprehensive understanding of the subject.

### Implications for clinical practice and future research

The findings suggest that rapid microbiological tests can alleviate clinical uncertainty and concerns about adverse outcomes while helping to manage patient expectations by reinforcing the aetiology of the infectious disease. Professionals, in general, favour integrating these rapid microbiological POCTs for managing respiratory tract infections with other POCTs, such as CRP, and other complementary strategies like delayed antibiotic prescribing and communication skills training. Future research should focus on optimising these strategies to minimise the burden on clinicians.

The results of this study should be considered when implementing rapid tests in primary care practices. Two specific aspects emerged during the study. First, these tests may be more useful in older adults, as they can identify specific viral and bacterial infections of interest in this age group. The association between certain viral infections and more severe outcomes in older individuals has been established.^23^ Understanding the perspectives of nursing staff in long-term care facilities in future quality studies could provide further insight into this issue. Similarly, it is important to consider patients’ opinions about these tests, as this could improve communication during consultations with professionals.

In conclusion, primary care healthcare professionals expressed both favourable and unfavourable opinions regarding the use of rapid respiratory tract infection tests. Unless the molecular tests reduce their price and time allocated for obtaining a result, their use will be limited. The burden pointed out by professionals regarding the routine utilisation of these tests is another aspect that must be tackled before implementing these in the primary care consultations. One potential solution could be to safely incorporate rapid tests as a triage tool to reduce clinical workload. Providing clear guidance and training on the use of these tests is crucial to ensure they are used in conjunction with clinical reasoning, helping to ease concerns about accurately interpreting rapid test results.

## Data Availability

Data are available upon reasonable request.
