# Supplementary material for: Views of Spanish primary care professionals on respiratory tract infection testing: a qualitative study
Source: BMJ Open. 2025 Sep 8;15(9):e102476. doi: 10.1136/bmjopen-2025-102476 (PMC12421173; doi:10.1136/bmjopen-2025-102476)
Supplement: Supplementary file 1 [file bmjopen-2025-102476.reviewer_comments.pdf]

PEER REVIEW HISTORY

BMJ Open publishes all reviews undertaken for accepted manuscripts. Reviewers are asked to complete a checklist review form and are provided with free text boxes to elaborate on their assessment. These free text comments are reproduced below.

ARTICLE DETAILS

Title (Provisional)

Views of Spanish primary care professionals on respiratory tract infection testing. A qualitative study.

Authors

Moragas, Ana; Cots, Josep M; Llor, Carl

VERSION 1 - REVIEW

**Reviewer**	**1**
**Name**	**Iramiot, Jacob**
**Affiliation**	**Busitema University, Microbiology and Immunology**
**Date**	**11-Apr-2025**
**COI**	**None**

---

consistency and the quality assurance mechanism are the major challenges in this paper

**** The reviewer provided a marked copy with additional comments. Please contact the publisher for full details. ****

**Reviewer**	**2**
**Name**	**Brown, Emily**
**Affiliation**	**University of Bristol, Centre for Academic Primary Care**
**Date**	**01-Jun-2025**
**COI**	**None**

Thank you for the opportunity to review this paper, which is well written and addresses an important and topical research area. I have the following comments:

Title/abstract

1. The title and abstract focuses on microbiological POCTs whereas quite a lot of the manuscript also reports on host response POCTs (e.g. CRP) and the comparison between these two types of POCTs. Perhaps title/abstract/objectives might be adjusted to better reflect this?

Introduction

1. The paper distinguishes molecular vs antigenic microbiological POCTs – I think it might be helpful to define each of these in the introduction. Molecular feels a very broad term, I'm more familiar with microbiological POCTs being defined as using nucleic acid or antigen-based tests.

Methods

1. Could you clarify if focus groups or semi structured interviews were used as both are mentioned. Why was the chosen approach taken and what are the strengths/limitations. I think it is focus groups but I'm a bit unclear from the manuscript.

2. How were participants recruited?

3. There is limited information about what questions were asked and how these questions were developed. Please could this be described in more detail in the manuscript and the topic guide shared as supplementary information.

4. The PPI statement talks about the study not interviewing patients and that a separate study interview patients in planned. This is not my understanding of what PPI is, and I believe that PPI is relevant to a study that interviews clinicians. Could the authors comment on whether there has been any PPI involvement?

5. What experience did the clinicians interview have of using these tests? It's stated in the paper that there is limited use of rapid microbiological POCTs and given this qualitative study isn't part of a wider study assessing the use of such tests, I'm wondering how much experience the clinicians interview have of using these tests. I think this is very important context to understanding the significance of the clinician perceptions presented here. Could this be added as a column in table 1 and discussed in the manuscript.

6. I think it would also be useful to highlight that there are many different microbiological POCTs e.g. nucleic acid vs antigens, single organism vs multiplex, and how long they take to give results. So the clinician experience will presumably be quite broad so they might not all be discussing similar types of tests. This could also be reflected in table 1 e.g. how regularly they use rapid microbiological POCTs in their clinical practice, for how many years, what types of tests, the range of times taken to get results from these tests, whether they were using the tests in the context of a study or as 'real life'. This will allow the reader to understand the context from which the clinicians speak.

7. The non expert group largely consists of trainees (only 2 qualified clinicians) with a limited number of years experience, so I wonder if they have much experience using these tests or if there comments are more hypothetical? I wonder if holding non expert focus groups with doctors/nurses who are qualified and have more years experience would be useful and add meaningfully to the data.

8. Were both transcripts were double coded?

9. How was reflexivity embedded in the study?

10. I wonder if two focus groups if enough to reach data saturation? I'm more familiar with 4-8 focus groups being reporting.

11. Were incentives offered for participation?

12. Why was thematic analysis chosen?

13. Could you report on the level of deprivation of the sites where the clinicians work?

14. Apologies if I have missed any of this but I don't think the following COREQ checklist items are clearly reported in the manuscript:

a. What was their occupation at the time of the study?

b. What experience or training did the researcher have?

c. Was a relationship established prior to study commencement?

d. What did the participants know about the researcher? e.g. personal goals, reasons for doing the research

e. What characteristics were reported about the inter viewer/facilitator? e.g. Bias, assumptions, reasons and interests in the research topic

f. How were participants selected? e.g. purposive, convenience, consecutive, snowball

g. How were participants approached? e.g. face-to-face, telephone, mail, email

h. How many people refused to participate or dropped out? Reasons?

i. Were questions, prompts, guides provided by the authors? Was it pilot tested?

j. Did the research use audio or visual recording to collect the data?

k. Were field notes made during and/or after the inter view or focus group?

l. Was data saturation discussed?

m. Were transcripts returned to participants for comment and/or correction?

n. Did authors provide a description of the coding tree?

o. Did participants provide feedback on the findings?

15. I don't think the COREQ checklist page numbers are correct e.g. "What experience or training did the researcher have?" says page 5, which is the introduction

Results

1. There seems to be a suggestion in the text the authors have written that host response POCTs (e.g. CRP) are useful for prognosis but not for diagnosis. I don't think this is always the case and host response POCTs are used diagnostically too. I think it would be useful to make it clear if that is what the authors belief (and if so perhaps provide a reference) or if that was the expressed views of the participants. If the latter some critique of this perception might be useful.

2. There's also a suggestion running through the text that if clinicians know the patient has a bacterial infection then they know antibiotics are needed. I think it would be interesting to know if clinicians discussed this in the interviews and there were

dissonant views around needing to know the prognosis rather than the diagnosis to guide antibiotic prescription?

3. I wonder if more comparison of the different views and any dissonance between the NE and E groups might be valuable. For example, some sections of the results only have quotes from the E group – were these views held by the NE group as well?

4. Were the limitation of microbiological POCTs discussed - either asked in the topic guide or raised by the clinicians? For example, microbiological POCTs are swab based and so results don't necessarily reflect causative infection in the respiratory tract e.g. commensal group A strep. I think it would be important to discuss whether clinicians raised this or not.

5. Page 10, line 41 – is there a reference for this statement? Or is it a patient perception, in which case this should be clarified

Discussion

1. I'm unclear of the novelty in these findings compared to reference 16, perhaps this could be better highlighted?

2. P14 line 42 and line 47 and P15 line 18 – reference 16 also reports these findings. Authors should note that this has already been report and that their study found similar.

3. In contrast to this study, reference 16 was conducted in the context of a trial of a rapid microbiological POCT, so all clinicians interviewed had experience of this test and they were all discussing the same test. This is not the case in this study and I have mentioned the potential limitations of this study in that regard above. However, I also think this could be highlighted as a positive of this study because clinicians here are likely reporting 'real life' use of POCTs rather than in the context of a study.

4. Page 16 line 29-30: "as they can more easily identify specific viral and bacterial infections of interest in this age group". I'm unclear of the rational behind this statement, is there a reference?

Reviewer 3

Name Chini, Gabriele

Affiliation Trento Provincial Authority for Health Services, Risorse Umane - Polo universitario delle Professioni Sanitarie di Trento

Date 10-Jun-2025

COI None

The authors conduct a much needed and interesting qualitative review.

I have some minor comments presented below

Thank you for this interesting paper, it was a pleasure to revise it. In my opinion it is very innovative and can support healthcare professional in their clinical practice. I have a few minor comments below.

ABSTRACT
Please, in introduction section Specify the type of qualitative research

INTRODUCTION
Please, provide in method section, a brief description of World Health Organization sources on actions to combat antibiotic resistance.

METHODS
This section is very clear and complete.
Provide a brief description of the guiding questions of the semi-structured interviews.
Specify the type of sampling and exclusion criteria, if any.
Indicate the response rate to invitations to participate in focus groups.

RESULTS
This section is very well described with significant themes, The quotes provided support the thematic analysis presented. Only few suggestions.
It is not always clear in which topics there was more or different input from experts and nonexperts
In table 2, Please, highlight the need for the criteria for performing the test and that it does not replace the clinical examination.

DISCUSSION
This is a very interesting section. The main implication for future research in this field are present.
The study found that the elderly population is the one in which these tests could be particularly useful; there is no mention of the palliative care, where the dilemma of whether or not to start antibiotic therapy often arises at the end of life. This study comes at a time of primary care system reform in which new community-based facilities may play an important role as referral centers for performing these tests

---

VERSION 1 - AUTHOR RESPONSE

We are delighted to have the opportunity to revise our paper in response to the comments provided by the three reviewers. We sincerely acknowledge the value of their feedback, which has enabled us to significantly improve the study. We have addressed all of the reviewers' queries and have itemised our responses to each point below, organized according to the relevant sections of the manuscript.

Title/abstract

Reviewer 1. Modify the title to 'respiratory tract infection tests'.

RESPONSE: We have changed the title as suggested.

Reviewer 1. In the Results section of the abstract, the authors need to clarify if this study was about rapid molecular tests or rapid microbiological tests.

RESPONSE: We have changed it accordingly.

Reviewer 2. The title and abstract focuses on microbiological POCTs whereas quite a lot of the manuscript also reports on host response POCTs (e.g. CRP) and the comparison between these two types of POCTs. Perhaps title/abstract/objectives might be adjusted to better reflect this?

RESPONSE: We have changed the title as suggested as follows: 'Views of primary care professionals on respiratory tract infection tests. A qualitative study.'

Reviewer 3. Please, in the introduction section Specify the type of qualitative research.

RESPONSE: We have changed it accordingly, as follows: 'Qualitative study design applying semi-structured interviews in two focus groups.'

Strengths and limitations

Reviewer 1. Second bullet point. The study targeted the healthcare workers views; how is not recruiting the patients a weakness?

RESPONSE: The reviewer is right. We have reformulated this sentence in the new version. The clinicians' belief that patients would be more interested in knowing the aetiology rather than the prognosis of the infection should be further investigated through patient inclusion.

Reviewer 1. Second bullet point. The authors seem to indicate the rapid diagnostics indicate the prognosis of the RTIs which is wrong.

RESPONSE: We have rephrased this bullet point as follows: 'Clinicians believe that patients are generally more interested in undergoing tests that identify the aetiology of an infection rather than those that provide information about its likely course or prognosis. Therefore, research involving patients should be encouraged to better understand their actual preferences and priorities regarding diagnostic testing.'

Reviewer 1. Third bullet point. It would be good to know how the authors decided that this was enough data.

RESPONSE: Thanks for the comment. We have rephrased it as follows: 'Interviews were conducted until data saturation was reached, ensuring that well-founded themes could be identified.'

Introduction

Reviewer 2. The paper distinguishes molecular vs antigenic microbiological POCTs – I think it might be helpful to define each of these in the introduction. Molecular feels a very broad term, I'm more familiar with microbiological POCTs being defined as using nucleic acid or antigen-based tests.

RESPONSE: We have defined this, as suggested by the reviewer, in the last paragraph of the Introduction section.

Reviewer 3. Please, provide a brief description of World Health Organization sources on actions to combat antibiotic resistance.

RESPONSE: We have added a paragraph in the Introduction highlighting this point and included the corresponding reference, as follows:

'In April 2025, the World Health Organization expanded its primary health care framework for addressing antimicrobial resistance. Key priorities include the integration of antimicrobial resistance into national health policies and primary care systems, the implementation of antimicrobial stewardship programs, and the promotion of community- and school-based antimicrobial resistance education campaigns. Additionally, a new community platform was launched to facilitate the sharing of best practices [5].'

Methods

<u>Reviewer 1</u>. Last paragraph before Ethical aspects. The authors do not mention any quality assurance mechanisms put in place right from the point of interviews and quality of transcripts.

RESPONSE: The interviews were video-recorded with the oral consent of participants in the online focus group and written consent from those participating in the in-person group. The recordings were transcribed by the two lead authors using computer-assisted transcription software (Google Docs Voice Typing).

<u>Reviewer 2</u>. Could you clarify if focus groups or semi structured interviews were used as both are mentioned. Why was the chosen approach taken and what are the strengths/limitations. I think it is focus groups but I'm a bit unclear from the manuscript.

RESPONSE: Yes, we conducted two focus groups and used a semi-structured interview guide to facilitate the discussions. While both focus groups and semi-structured interviews are mentioned in the manuscript, we can clarify that the data was collected specifically through focus group discussions. The semi-structured guide served as a framework to ensure consistency across the sessions while still allowing for open-ended responses and organic discussion among participants.

<u>Reviewer 2</u>. How were participants recruited?

RESPONSE: The Expert group was recruited via email. An initial message was sent explaining the purpose of the meeting, followed by a reminder with the date and time. All participants responded, with only one unable to attend due to a scheduling conflict. Participants in the Non-Expert group were recruited during the Trainee Day event at the primary health centre. Researcher AM explained the purpose of the study, and all participants agreed to take part and signed a written consent form. We have expanded on this in the Methods section.

<u>Reviewer 2</u>. There is limited information about what questions were asked and how these questions were developed. Please could this be described in more detail in the manuscript and the topic guide shared as supplementary information.

RESPONSE: We have added the topiv guide as an appendix to the article (Supplemental Table 1).

<u>Reviewer 2</u>. The PPI statement talks about the study not interviewing patients and that a separate study interview patients in planned. This is not my understanding of what

PPI is, and I believe that PPI is relevant to a study that interviews clinicians. Could the authors comment on whether there has been any PPI involvement?

RESPONSE: We believe it is important to understand patients' perspectives when implementing a new rapid diagnostic technique in primary care consultations. It is valuable to explore their beliefs and whether they think such tests could be useful or not.

Reviewer 2. What experience did the clinicians interview have of using these tests? It's stated in the paper that there is limited use of rapid microbiological POCTs and given this qualitative study isn't part of a wider study assessing the use of such tests, I'm wondering how much experience the clinicians interview have of using these tests. I think this is very important context to understanding the significance of the clinician perceptions presented here. Could this be added as a column in table 1 and discussed in the manuscript.

RESPONSE: Thank you for the comment. We have added this information to Table 1.

Reviewer 2. I think it would also be useful to highlight that there are many different microbiological POCTs e.g. nucleic acid vs antigens, single organism vs multiplex, and how long they take to give results. So the clinician experience will presumably be quite broad so they might not all be discussing similar types of tests. This could also be reflected in table 1 e.g. how regularly they use rapid microbiological POCTs in their clinical practice, for how many years, what types of tests, the range of times taken to get results from these tests, whether they were using the tests in the context of a study or as 'real life'. This will allow the reader to understand the context from which the clinicians speak.

RESPONSE: We have added this information to Table 1. We have indicated whether the tests have been in use for more or less than five years. It is very difficult to determine how frequently the tests are used, as just as mentioned it reflects real-life practice. There are days or times of the year when the tests are used more frequently, and other periods, such as summer in our area, when respiratory infections decrease significantly and the tests are rarely used.

Reviewer 2. The non expert group largely consists of trainees (only 2 qualified clinicians) with a limited number of years experience, so I wonder if they have much experience using these tests or if there comments are more hypothetical? I wonder if holding non expert focus groups with doctors/nurses who are qualified and have more years experience would be useful and add meaningfully to the data.

RESPONSE: The NE participants have a limited number of years' experience, but we think it's a really relevant point of view because they go in different services at the hospital, at the primary care, at the emergencies… and they know different realities, with different experiences.

Reviewer 2. Were both transcripts were double coded?

RESPONSE: The first focus group with the expert group was double coded to enhance the rigour of the analysis. However, the session with the non-expert group was not double coded, as it was conducted solely by the moderator. In this case, double coding was not feasible.

Reviewer 2. How was reflexivity embedded in the study?

RESPONSE: The research team maintained reflexive awareness throughout the study, acknowledging their professional backgrounds and potential biases. Reflective memos were kept to monitor the influence of researcher perspectives on data collection and analysis.

Reviewer 2. I wonder if two focus groups if enough to reach data saturation? I'm more familiar with 4-8 focus groups being reporting.

RESPONSE: Thank you very much for your message. In our study, we are confident that data saturation was achieved with the two focus groups conducted. However, we recognize the value of further exploration and there are plans to extend this research in the future by including additional groups. These will involve pediatricians, patients, and doctors from other settings such as hospitals, which we believe will provide a broader and more comprehensive understanding of the topic.

Reviewer 2. Were incentives offered for participation?

RESPONSE: We did not provide any incentives to participants for their time.

Reviewer 2. Why was thematic analysis chosen?

RESPONSE: Thematic analysis was selected as the method for analysing the qualitative data because it is a flexible and widely used approach. Given the exploratory nature of this study, aiming to understand healthcare professionals' views on rapid microbiological tests in primary care, this method enables a rich, detailed, and nuanced understanding of participants' perspectives. In addition, thematic analysis is particularly well suited for studies with focus groups because it can accommodate varied and complex data, allowing researchers to capture both explicit and implicit meanings within participants' accounts. Additionally, it provides a transparent and rigorous framework for coding and theme development, which supports the credibility and trustworthiness of the findings.

Reviewer 2. Could you report on the level of deprivation of the sites where the clinicians work?

RESPONSE: We have not included a deprivation level because all healthcare professionals work in a medium or slightly low socio-economic environment, without many differences between them. However, we have distinguished between urban and rural centers and between continuous care centers and health centers. Nevertheless, we have indeed distinguished between urban and rural centers, and between primary health centres and emergency departments.

Reviewer 2. Apologies if I have missed any of this but I don't think the following COREQ checklist items are clearly reported in the manuscript:

a. What was their occupation at the time of the study?

RESPONSE: CL is currently working as a researcher at the Jordi Gol Institute for Research, and is affiliated with CIBER as well as the University of Southern Denmark.

Researcher AM works as a general practitioner at a primary care centre, is an associate professor at Rovira i Virgili University, and collaborates as a researcher at the Jordi Gol Institute for Research. The affiliation is included on both pages 1 and 6.

b. What experience or training did the researcher have?

RESPONSE: Both researchers have participated in several studies, both quantitative and qualitative, in the field of infections in primary care.

b. Was a relationship established prior to study commencement?
RESPONSE: The researchers have collaborated on previous studies with some of the participants.

d. What did the participants know about the researcher? e.g. personal goals, reasons for doing the research.

RESPONSE: Some participants from the Expert group were already familiar with the researchers, as they have been involved in the Spanish Sociey of Family Medicine research group and have collaborated on previous studies. Some participants from the Non-Expert group knew the researcher because they work in nearby primary care centres. However, other participants had no prior connection with the research team.

e. What characteristics were reported about the inter viewer/facilitator? e.g. Bias, assumptions, reasons and interests in the research tòpic

RESPONSE: The participants from the Expert group knew the researchers beforehand because they had participated in the Spanish Society of Family Medicine research group and had previously conducted joint studies. The participants from the Non-Expert group knew the researcher as they work in nearby primary care centres.

f. How were participants selected? e.g. purposive, convenience, consecutive, snowball

RESPONSE: We employed a convenience sampling strategy, selecting participants based on their availability and relevance to the study context.

g. How were participants approached? e.g. face-to-face, telephone, mail, email

RESPONSE: As mentioned in this new version in the Methods section, participants in the Expert group were contacted via email. In contrast, participants in the Non-Expert group were approached in person during a training day held at their primary care centres.

h. How many people refused to participate or dropped out? Reasons?

RESPONSE: We have also added in this revised version that one of the invited participants was unable to take part due to scheduling conflicts.

i. Were questions, prompts, guides provided by the authors? Was it pilot tested?

RESPONSE: The authors conducted a review of the relevant literature on point-of-care testing and antibiotic prescribing in primary care in order to inform the development of the interview guide. This process ensured that the questions were grounded in existing evidence and addressed key themes relevant to the study objectives. A formal pilot test of the guide was not conducted; however, the guide was reviewed and refined collaboratively by the research team to ensure clarity, coherence, and appropriateness.

j. Did the research use audio or visual recording to collect the data?

RESPONSE: The E group was conducted online and both audio and visual recordings were made. The NE group was conducted face-to-face and an audio recording was made.

k. Were field notes made during and/or after the interview or focus group?

RESPONSE: During the focus groups, the researchers took detailed observational notes to capture non-verbal cues, group dynamics, and contextual factors that might not be fully reflected in the audio recordings. These notes were subsequently shared among the research team and discussed in debriefing sessions following each interview. This process helped enrich the interpretation of the data, ensured consistency in the analysis, and contributed to the reflexive approach adopted throughout the study.

l. Was data saturation discussed?

RESPONSE: Data saturation was assessed collaboratively by the research team after the focus group sessions.

m. Were transcripts returned to participants for comment and/or correction?

RESPONSE: The transcripts, coding tree description, and feedback were shared with a select group of participants only.

n. Did authors provide a description of the coding tree?

RESPONSE: The transcripts, the description of the coding tree, and the feedback were shared only with some of the participants.

o. Did participants provide feedback on the findings?

RESPONSE: The transcripts, the description of the coding tree, and the feedback were shared only with some of the participants.

Reviewer 2. I don't think the COREQ checklist page numbers are correct e.g. "What experience or training did the researcher have?" says page 5, which is the introduction.

RESPONSE: The information is on page 6.

Reviewer 3. This section is very clear and complete. Provide a brief description of the guiding questions of the semi-structured interviews. Specify the type of sampling and exclusion criteria, if any. Indicate the response rate to invitations to participate in focus groups.

RESPONSE: The Expert (E) group was recruited via email. An initial message was sent to explain the purpose of the meeting, followed by a reminder that included the date and time. All invited participants responded, with only one unable to attend due to scheduling conflicts. Participants in the Non-Expert (NE) group were recruited in person during the Trainee Day event held at the primary health centre. Researcher AM explained the objectives of the study directly to the attendees, and all agreed to participate.

Results

Reviewer 2. There seems to be a suggestion in the text the authors have written that host response POCTs (e.g. CRP) are useful for prognosis but not for diagnosis. I don't think this is always the case and host response POCTs are used diagnostically too. I think it would be useful to make it clear if that is what the authors belief (and if so

perhaps provide a reference) or if that was the expressed views of the participants. If the latter some critique of this perception might be useful.

RESPONSE: There is a direct question posed to the professionals asking whether they prefer tests that provide prognostic information or those that offer diagnostic results. This question helps to capture and establish the different perspectives among participants regarding the utility and value of each type of test.

Reviewer 2. There's also a suggestion running through the text that if clinicians know the patient has a bacterial infection then they know antibiotics are needed. I think it would be interesting to know if clinicians discussed this in the interviews and there were dissonant views around needing to know the prognosis rather than the diagnosis to guide antibiotic prescription?

RESPONSE: This topic is also discussed in response to a direct question posed to the participants regarding their preference for diagnostic tests versus prognostic tests. The discussion that follows helps to explore and contrast their views on the relative benefits and applications of each type of test in clinical practice.

Reviewer 2. I wonder if more comparison of the different views and any dissonance between the NE and E groups might be valuable. For example, some sections of the results only have quotes from the E group – were these views held by the NE group as well?

RESPONSE: We believe that the Non-Expert (NE) group has less experience, which may make it more challenging for them to respond fully to these questions. This difference in experience could explain some of the uncertainties or hesitations observed during their responses. We hope this helps to clarify any doubts regarding their engagement with the topics discussed.

Reviewer 2. Were the limitation of microbiological POCTs discussed - either asked in the topic guide or raised by the clinicians? For example, microbiological POCTs are swab based and so results don't necessarily reflect causative infection in the respiratory tract e.g. commensal group A strep. I think it would be important to discuss whether clinicians raised this or not.

RESPONSE: Yes, the limitations of microbiological POCTs were indeed discussed during the focus groups. Although the topic guide did not explicitly include a question on this aspect, several clinicians raised concerns spontaneously regarding the interpretation of test results. They noted that positive results might not always indicate a causative infection, as some detected organisms could be commensal or colonizers rather than pathogens, mainly in mixed bacterial-viral infections. This was mainly raised during the post-sesson reflection. Although initial thematic saturation was reached during the focus groups, post-session reflection provided this particular insight, enriching the analysis. However, we did not include it, as it did not emerge directly from the focus group discussions.

Reviewer 2. Page 10, line 41 – is there a reference for this statement? Or is it a patient perception, in which case this should be clarified.

RESPONSE: Yes, we have included a reference to further illustrate the concept of overutilization of POCTs in respiratory tract infections, specifically drawing on evidence

from Scandinavian countries. This addition helps to contextualize the clinicians' concerns about potential overuse and misinterpretation of test results.

Reviewer 3. This section is very well described with significant themes, The quotes provided support the thematic analysis presented. Only few suggestions. It is not always clear in which topics there was more or different input from experts and nonexperts In table 2, Please, highlight the need for the criteria for performing the test and that it does not replace the clinical examination.

RESPONSE: The reviewer is absolutely right. To clarify this point, we have added a statement in Table 2 emphasising that the use of tests does not replace the need for a thorough medical history and physical examination. This addition aims to reinforce the importance of comprehensive clinical assessment alongside diagnostic testing.

Discussion

Reviewer 1. First paragraph. When you mention 'microbiological tests and CRP testing as complementary, non-experts tend to prefer', do you refer to rapid or conventional tests?

RESPONSE: Thank you for the comment. This refers to rapid microbiological tests. It has been added to the text in the revised version.

Reviewer 1. Second paragraph, comparison with existing research findings: There should be consistency so that you do not confuse rapid microbiology tests and conventional microbiology tests.

RESPONSE: The reviewer is right, and we have revised the text accordingly to clarify this point. We have now explicitly added the term rapid microbiological tests in the updated version to ensure clarity for the reader.

Reviewer 1. Implications for clinical research and future research. When you are mentioning, the findings suggest that microbiological tests, are you referring to rapid or conventional tests?

RESPONSE: It is true, and we have updated the text accordingly by adding the term rapid microbiological tests.

Reviewer 1. Last paragraph. Again, was tis study on microbiology, molecular biology, molecular microbiological or rapid microbiology?

RESPONSE: Thank you again for the comment. You are right, and we have revised the text accordingly by adding the term rapid microbiological tests.

Reviewer 2. I'm unclear of the novelty in these findings compared to reference 16, perhaps this could be better highlighted?

RESPONSE: We have changed the reference it refers to. It is now correct. Thank you for the comment.

Reviewer 2. 2. P14 line 42 and line 47 and P15 line 18 – reference 16 also reports these findings. Authors should note that this has already been report and that their study found similar.

RESPONSE: Thank you for the comment. We have clarified this point in the revised version as follows: 'Similar to the findings of the British study [16], our study also emphasises that the use of rapid microbiological tests can play a valuable role in managing patient expectations, particularly by providing greater clarity about the cause of infection. However, this benefit may come with certain risks. If clinicians begin to over-rely on these tests —using them prior to a thorough clinical examination or treating them as standalone diagnostic tools— it may undermine essential clinical skills and judgement. This concern has also been noted in relation to CRP rapid testing in some Scandinavian countries [17].'

Reviewer 2. In contrast to this study, reference 16 was conducted in the context of a trial of a rapid microbiological POCT, so all clinicians interviewed had experience of this test and they were all discussing the same test. This is not the case in this study and I have mentioned the potential limitations of this study in that regard above. However, I also think this could be highlighted as a positive of this study because clinicians here are likely reporting 'real life' use of POCTs rather than in the context of a study.

RESPONSE: You are absolutely right. In our study, clinicians are reflecting on their experiences with POCTs as they are used in real-life settings, within their everyday clinical practice at actual healthcare centres. While we acknowledge the limitations of this approach, we also believe it adds value by capturing the complexity, variability, and practical challenges of implementing POCTs in real-world primary care environaments.

Reviewer 2. Page 16 line 29-30: "as they can more easily identify specific viral and bacterial infections of interest in this age group". I'm unclear of the rational behind this statement, is there a reference?

RESPONSE: Older individuals often present with atypical or less specific symptoms of infection, making diagnosis more challenging. In this context, the ability of POCTs to provide timely and accurate microbiological information can aid in distinguishing between viral and bacterial etiologies, thereby supporting more targeted treatment decisions and helping to avoid unnecessary antibiotic use. In addition, the association between certain viral infections, such as influenza, respiratory syncytial virus and COVID-19, and an increased risk of severe complications, hospitalisation, and mortality in older adults is well documented. Therefore, timely identification of these pathogens through POCTs could contribute to improved clinical outcomes by facilitating early intervention and appropriate patient management. We have added an additional reference to better illustrate this idea and to support the relevance of using rapid microbiological tests in older adults.

Reviewer 3. This is a very interesting section. The main implication for future research in this field are present. The study found that the elderly population is the one in which these tests could be particularly useful; there is no mention of the palliative care, where the dilemma of whether or not to start antibiotic therapy often arises at the end of life. This study comes at a time of primary care system reform in which new community-based facilities may play an important role as referral centers for performing these tests.

RESPONSE: reviewer is right in noting that, in palliative care patients, the dilemma of whether or not to use antibiotics often arises at the end of life. However, this specific patient group was not considered in the present study.

We hope that this study has suitably improved to be accepted for publication in your prestigious journal

---

VERSION 2 - AUTHOR RESPONSE

Reviewer: 2

Dr. Emily Brown, University of Bristol

Comments to the Author:

Thank you for the opportunity to review this paper, which is well written and addresses an important and topical research area. I have the following comments:

Title/abstract

1. The title and abstract focuses on microbiological POCTs whereas quite a lot of the manuscript also reports on host response POCTs (e.g. CRP) and the comparison between these two types of POCTs. Perhaps title/abstract/objectives might be adjusted to better reflect this?

RESPONSE: We have changed the title as suggested as follows: 'Views of Spanish primary care professionals on respiratory tract infection testing. A qualitative study.'

Introduction

1. The paper distinguishes molecular vs antigenic microbiological POCTs – I think it might be helpful to define each of these in the introduction. Molecular feels a very broad term, I'm more familiar with microbiological POCTs being defined as using nucleic acid or antigen-based tests.

RESPONSE: We have defined this, as suggested by the reviewer, in the last paragraph of the Introduction section.

Methods

1. Could you clarify if focus groups or semi structured interviews were used as both are mentioned. Why was the chosen approach taken and what are the strengths/limitations. I think it is focus groups but I'm a bit unclear from the manuscript.

RESPONSE: Yes, we conducted two focus groups and used a semi-structured interview guide to facilitate the discussions. Although both focus groups and semi-structured interviews are mentioned in the manuscript, we would like to clarify that the data was specifically collected through focus group discussions. The semi-structured guide provided a framework to ensure consistency across sessions, while still allowing for open-ended responses and discussions among participants.

2. How were participants recruited?

RESPONSE: The Expert group was recruited via email. An initial message explained the purpose of the meeting, followed by a reminder with the date and time. All

participants responded, with only one unable to attend due to a scheduling conflict. Participants in the Non-Expert group were recruited during the Trainee Day event at the primary health centre. Researcher AM explained the purpose of the study, and all participants agreed to take part and signed written consent forms. We have expanded on this in the Methods section.

3. There is limited information about what questions were asked and how these questions were developed. Please could this be described in more detail in the manuscript and the topic guide shared as supplementary information.

RESPONSE: The authors AM and CL developed the list of questions based on a comprehensive literature review, which served as a guide to initiate discussions in both focus groups. In accordance with the editor's instructions, we have included the topic guide as a table in the manuscript (Table 2).

4. The PPI statement talks about the study not interviewing patients and that a separate study interview patients in planned. This is not my understanding of what PPI is, and I believe that PPI is relevant to a study that interviews clinicians. Could the authors comment on whether there has been any PPI involvement?

RESPONSE: We acknowledge the importance of understanding patients' perspectives when implementing new rapid diagnostic techniques in primary care consultations. Exploring patients' beliefs and perceptions regarding the usefulness of such tests is indeed valuable. However, no patients were involved in this study; accordingly, we have removed this information from the manuscript.

5. What experience did the clinicians interview have of using these tests? It's stated in the paper that there is limited use of rapid microbiological POCTs and given this qualitative study isn't part of a wider study assessing the use of such tests, I'm wondering how much experience the clinicians interview have of using these tests. I think this is very important context to understanding the significance of the clinician perceptions presented here. Could this be added as a column in table 1 and discussed in the manuscript.

RESPONSE: Thank you for the comment. We have added this information to Table 1.

6. I think it would also be useful to highlight that there are many different microbiological POCTs e.g. nucleic acid vs antigens, single organism vs multiplex, and how long they take to give results. So the clinician experience will presumably be quite broad so they might not all be discussing similar types of tests. This could also be reflected in table 1 e.g. how regularly they use rapid microbiological POCTs in their clinical practice, for how many years, what types of tests, the range of times taken to get results from these tests, whether they were using the tests in the context of a study or as 'real life'. This will allow the reader to understand the context from which the clinicians speak.

RESPONSE: We have added this information to Table 1. We have indicated whether the tests have been in use for more or less than five years. It is very difficult to determine how frequently the tests are used, as just as mentioned it reflects real-life practice. There are days or times of the year when the tests are used more frequently, and other periods, such as summer in our area, when respiratory infections decrease significantly and the tests are rarely used.

7. The non expert group largely consists of trainees (only 2 qualified clinicians) with a limited number of years experience, so I wonder if they have much experience using these tests or if there comments are more hypothetical? I wonder if holding non expert focus groups with doctors/nurses who are qualified and have more years experience would be useful and add meaningfully to the data.

RESPONSE: Although the Non-Expert (NE) participants have a limited number of years of professional experience, their perspectives are highly relevant. They rotate through various departments, including hospital services, primary care, and emergency care, which provides them with exposure to diverse clinical settings and a broad range of experiences.

8. Were both transcripts were double coded?

RESPONSE: The first focus group with the expert group was double coded to enhance the rigour of the analysis. However, the session with the non-expert group was not double coded, as it was conducted solely by the moderator, making double coding unfeasible. This clarification has been added to the manuscript as suggested by the editor.

9. How was reflexivity embedded in the study?

RESPONSE: The research team maintained reflexive awareness throughout the study by acknowledging their professional backgrounds and potential biases. Reflective memos were documented to monitor and address the influence of researcher perspectives on data collection and analysis. This information has been incorporated into the manuscript.

10. I wonder if two focus groups if enough to reach data saturation? I'm more familiar with 4-8 focus groups being reporting.

RESPONSE: Thank you very much for your message. In our study, we are confident that data saturation was achieved with the two focus groups conducted. However, we recognize the value of further exploration and there are plans to extend this research in the future by including additional groups. These will involve pediatricians, patients, and doctors from other settings such as hospitals, which we believe will provide a broader and more comprehensive understanding of the topic.

11. Were incentives offered for participation?

RESPONSE: We did not provide any incentives to participants for their time.

12. Why was thematic analysis chosen?

RESPONSE: Thematic analysis was selected as the method for analysing the qualitative data because it is a flexible and widely used approach. Given the exploratory nature of this study, aiming to understand healthcare professionals' views on rapid microbiological tests in primary care, this method enables a rich, detailed, and nuanced understanding of participants' perspectives. In addition, thematic analysis is particularly well suited for studies with focus groups because it can accommodate varied and complex data, allowing researchers to capture both explicit and implicit meanings within participants' accounts. Additionally, it provides a transparent and

rigorous framework for coding and theme development, which supports the credibility and trustworthiness of the findings. We have added this information to the article.

13. Could you report on the level of deprivation of the sites where the clinicians work?

RESPONSE: We have not included a deprivation level because all healthcare professionals work in a medium or slightly low socio-economic environment, without many differences between them. However, we have distinguished between urban and rural centers and between continuous care centers and health centers. Nevertheless, we have indeed distinguished between urban and rural centers, and between primary health centres and emergency departments.

14. Apologies if I have missed any of this but I don't think the following COREQ checklist items are clearly reported in the manuscript:

a. What was their occupation at the time of the study?

RESPONSE: Researcher AM works as a general practitioner at a primary care centre, is an associate professor at Rovira i Virgili University, and collaborates as a researcher at the Jordi Gol Institute for Research. CL is currently working as a researcher at the Jordi Gol Institute for Research, and is affiliated with CIBER as well as the University of Southern Denmark. The affiliation is included on both pages 1 and 6. We have added this information to the article.

b. What experience or training did the researcher have?

RESPONSE: Both researchers have participated in several studies, both quantitative and qualitative, in the field of infections in primary care.

c. Was a relationship established prior to study commencement?

RESPONSE: The researchers have collaborated on previous studies with some of the participants. We have added this information to the article.

d. What did the participants know about the researcher? e.g. personal goals, reasons for doing the research

RESPONSE: Some participants from the Expert group were already familiar with the researchers, as they have been involved in the Spanish Sociey of Family Medicine research group and have collaborated on previous studies. Some participants from the Non-Expert group knew the researcher because they work in nearby primary care centres. However, other participants had no prior connection with the research team. We have added this information to the article.

e. What characteristics were reported about the interviewer/facilitator? e.g. Bias, assumptions, reasons and interests in the research topic

RESPONSE: The participants from the Expert group knew the researchers beforehand because they had participated in the Spanish Society of Family Medicine research group and had previously conducted joint studies. The participants from the Non-Expert group knew the researcher as they work in nearby primary care centres. We have added this information to the article.

f. How were participants selected? e.g. purposive, convenience, consecutive, snowball

RESPONSE: We employed a convenience sampling strategy, selecting participants based on their availability and relevance to the study context.

g. How were participants approached? e.g. face-to-face, telephone, mail, email

RESPONSE: As mentioned in this new version in the Methods section, participants in the Expert group were contacted via email. In contrast, participants in the Non-Expert group were approached in person during a training day held at their primary care centres.

h. How many people refused to participate or dropped out? Reasons?

RESPONSE: We have also added in this revised version that one of the invited participants was unable to take part due to scheduling conflicts. This information is on page 6.

i. Were questions, prompts, guides provided by the authors? Was it pilot tested?

RESPONSE: The authors conducted a review of the relevant literature on point-of-care testing and antibiotic prescribing in primary care in order to inform the development of the interview guide. This process ensured that the questions were grounded in existing evidence and addressed key themes relevant to the study objectives. A formal pilot test of the guide was not conducted; however, the guide was reviewed and refined collaboratively by the research team to ensure clarity, coherence, and appropriateness.

j. Did the research use audio or visual recording to collect the data?

RESPONSE: The E group was conducted online and both audio and visual recordings were made. The NE group was conducted face-to-face and an audio recording was made.

k. Were field notes made during and/or after the interview or focus group?

RESPONSE: During the focus groups, the researchers took detailed observational notes to capture non-verbal cues, group dynamics, and contextual factors that might not be fully reflected in the audio recordings. These notes were subsequently shared among the research team and discussed in debriefing sessions following each interview. This process helped enrich the interpretation of the data, ensured consistency in the analysis, and contributed to the reflexive approach adopted throughout the study.

l. Was data saturation discussed?

RESPONSE: Data saturation was assessed collaboratively by the research team after the focus group sessions.

m. Were transcripts returned to participants for comment and/or correction?

RESPONSE: Transcripts were returned for review and comment to a subset of participants. This approach was adopted due to logistical challenges in reaching the entire participant pool, as detailed on page 6.

n. Did authors provide a description of the coding tree?

RESPONSE: A description of the coding tree was shared with a selected group of participants alongside the transcripts to obtain targeted feedback. This process is outlined on page 6.

o. Did participants provide feedback on the findings?

RESPONSE: Feedback on the findings, including the coding structure and transcripts, was obtained from a limited number of participants to accommodate challenges related to participant availability and dispersion. Further details are provided on page 6.

15. I don't think the COREQ checklist page numbers are correct e.g. "What experience or training did the researcher have?" says page 5, which is the introduction

RESPONSE: The information is on page 5.

Results

1. There seems to be a suggestion in the text the authors have written that host response POCTs (e.g. CRP) are useful for prognosis but not for diagnosis. I don't think this is always the case and host response POCTs are used diagnostically too. I think it would be useful to make it clear if that is what the authors belief (and if so perhaps provide a reference) or if that was the expressed views of the participants. If the latter some critique of this perception might be useful.

RESPONSE: There is a direct question posed to the professionals asking whether they prefer tests that provide prognostic information or those that offer diagnostic results. This question helps to capture and establish the different perspectives among participants regarding the utility and value of each type of test.

2. There's also a suggestion running through the text that if clinicians know the patient has a bacterial infection then they know antibiotics are needed. I think it would be interesting to know if clinicians discussed this in the interviews and there were dissonant views around needing to know the prognosis rather than the diagnosis to guide antibiotic prescription?

RESPONSE: This topic is also discussed in response to a direct question posed to the participants regarding their preference for diagnostic tests versus prognostic tests. The discussion that follows helps to explore and contrast their views on the relative benefits and applications of each type of test in clinical practice.

3. I wonder if more comparison of the different views and any dissonance between the NE and E groups might be valuable. For example, some sections of the results only have quotes from the E group – were these views held by the NE group as well?

RESPONSE: We recognize that the Non-Expert (NE) group has comparatively less experience, which may have limited their ability to respond comprehensively to certain questions. This difference in experience could account for some of the uncertainties or hesitations observed during their responses. We have acknowledged this as a limitation of the study to provide greater transparency regarding participant engagement with the discussed topics.

4. Were the limitation of microbiological POCTs discussed - either asked in the topic guide or raised by the clinicians? For example, microbiological POCTs are swab based and so results don't necessarily reflect causative infection in the respiratory tract e.g. commensal group A strep. I think it would be important to discuss whether clinicians raised this or not.

RESPONSE:. Yes, the limitations of microbiological POCTs were discussed during the focus groups. Although the topic guide did not explicitly include a question on this issue, several clinicians spontaneously raised concerns regarding the interpretation of test results. Specifically, they noted that positive results may not always indicate a causative infection, as some detected organisms could be commensal or colonizers—particularly in the context of mixed bacterial-viral infections. This concern was primarily identified during post-session reflection. While initial thematic saturation was reached during the focus groups, the post-session reflection provided this additional insight, thereby enriching the overall analysis. Although this theme did not emerge directly during the discussions, it has now been added to the Results section.

5. Page 10, line 41 – is there a reference for this statement? Or is it a patient perception, in which case this should be clarified

RESPONSE: Yes, we have included a reference to further illustrate the concept of overutilization of POCTs in respiratory tract infections, specifically drawing on evidence from Scandinavian countries. This addition helps to contextualize the clinicians' concerns about potential overuse and misinterpretation of test results.

Discussion

1. I'm unclear of the novelty in these findings compared to reference 16, perhaps this could be better highlighted?

RESPONSE: We have changed the reference it refers to. It is now correct. Thank you for the comment.

2. P14 line 42 and line 47 and P15 line 18 – reference 16 also reports these findings. Authors should note that this has already been report and that their study found similar.

RESPONSE: Thank you for the comment. We have clarified this point in the revised version as follows: 'Similar to the findings of the British study [16], our study also emphasises that the use of rapid microbiological tests can play a valuable role in managing patient expectations, particularly by providing greater clarity about the cause of infection. However, this benefit may come with certain risks. If clinicians begin to over-rely on these tests —using them prior to a thorough clinical examination or treating them as standalone diagnostic tools— it may undermine essential clinical skills and judgement. This concern has also been noted in relation to CRP rapid testing in some Scandinavian countries [17].'

3. In contrast to this study, reference 16 was conducted in the context of a trial of a rapid microbiological POCT, so all clinicians interviewed had experience of this test and they were all discussing the same test. This is not the case in this study and I have mentioned the potential limitations of this study in that regard above. However, I also think this could be highlighted as a positive of this study because clinicians here are likely reporting 'real life' use of POCTs rather than in the context of a study.

RESPONSE: In our study, clinicians are reflecting on their experiences with POCTs as they are used in real-life settings, within their everyday clinical practice at actual healthcare centres. While we acknowledge the limitations of this approach, we also believe it adds value by capturing the complexity, variability, and practical challenges of implementing POCTs in real-world primary care environanments.

4. Page 16 line 29-30: "as they can more easily identify specific viral and bacterial infections of interest in this age group". I'm unclear of the rational behind this statement, is there a reference?

RESPONSE: Older individuals often present with atypical or less specific symptoms of infection, making diagnosis more challenging. In this context, the ability of POCTs to provide timely and accurate microbiological information can aid in distinguishing between viral and bacterial etiologies, thereby supporting more targeted treatment decisions and helping to avoid unnecessary antibiotic use. In addition, the association between certain viral infections, such as influenza, respiratory syncytial virus and

COVID-19, and an increased risk of severe complications, hospitalisation, and mortality in older adults is well documented. Therefore, timely identification of these pathogens through POCTs could contribute to improved clinical outcomes by facilitating early intervention and appropriate patient management. We have added an additional reference to better illustrate this idea and to support the relevance of using rapid microbiological tests in older adults.

VERSION 3 - REVIEW

**Reviewer**	**2**
**Name**	**Brown, Emily**
**Affiliation**	**University of Bristol, Centre for Academic Primary Care**
**Date**	**13-Aug-2025**
**COI**	

Thank you again for this interesting study and thank you very much for taking the time to reply to my comments and update the manuscript. My comments are below.

Abstract

1. I think the following addition at the end of the objectives sentence would help clarify what the study involved “based on their experience in routine practice”

2. I think the conclusion may be better worded as “This study highlights how clinicians perceive respiratory tract infection tests to aid prescribe decisions amid uncertainty”

Methods

3. I think the description of the design is still somewhat confusing. E.g. “Abstract: Design. A qualitative study design was employed, using semi-structured interviews conducted in two focus group discussions.” I would suggest re-wording as: Design. A qualitative study design was employed. Two focus group discussions were conducted. I would tend to refer to the guide used for the FGDs as a topic guide rather than using the word ‘interviews’ as I think that is confusing. I would suggest updating the methods section accordingly too (i.e. don’t use the word interview – instead discuss the semi-structured topic guide)

4. I think there is still some confusion about the patient and public involvement – my understanding is this section is not about whether patients were recruited to the study, but whether there was any patient and public involvement e.g. input into the design of the study and interpretation of results etc.

5. Could the sample strategy be added to the manuscript e.g. the answer to my question about this question in the COREQ checklist (“convenience sampling strategy”)

Results

6. Thank you for including the topic guide. It seems some sections of the topic guide aren't reported e.g. the information clinicians would want about a POCT to feel confident introducing it. I think this would be very useful information to report.

7. I don't think this query has been answered "There seems to be a suggestion in the text the authors have written that host response POCTs (e.g. CRP) are useful for prognosis but not for diagnosis. I don't think this is always the case and host response POCTs are used diagnostically too. I think it would be useful to make it clear if that is what the authors belief (and if so perhaps provide a reference) or if that was the expressed views of the participants. If the latter some critique of this perception might be useful." I think the way that the question has been worded in the study suggests that CRP tests only provided prognostic information, not diagnostic information, which in my view isn't the case (e.g. CRP tests might be used to help distinguish viral from bacterial infection). Perhaps this could be discussed/referenced in the manuscript?

8. I don't follow the answer to my comment: "There's also a suggestion running through the text that if clinicians know the patient has a bacterial infection then they know antibiotics are needed. I think it would be interesting to know if clinicians discussed this in the interviews and there were dissonant views around needing to know the prognosis rather than the diagnosis to guide antibiotic prescription?"

9. Thank you for your reply to my comment "3. I wonder if more comparison of the different views and any dissonance between the NE and E groups might be valuable. For example, some sections of the results only have quotes from the E group – were these views held by the NE group as well?". I wonder if it would be value to report the uncertainties and hesitations that you mention of the NE group?

10. Thank you for your reply to my comment "5. Page 10, line 41 – is there a reference for this statement? Or is it a patient perception, in which case this should be clarified". I can't see that a reference has been added to this statement in the text of the results section? ("This issue is already occurring with CRP rapid testing in some Scandinavian countries.")

Discussion

11. I think a key limitation is the very little experience across both E and NE groups of using molecular tests – I think this should be reflected in the discussion, making it clear that most clinicians in the study only had experience of antigen tests.

12. Thank you for your reply to my comment "P14 line 42 and line 47 and P15 line 18 – reference 16 also reports these findings. Authors should note that this has already been report and that their study found similar." I think this change has been made for p14 line 42 but not for the other findings I noted similarity with reference 16.

13. I think it might be useful to emphasis the differences between this study and reference 16 (to emphasize the novelty of your findings). Firstly that reference 16 was conducted in the context of a trial of a rapid microbiological POCT, where as here clinicians are likely reporting 'real life' use of POCTs rather than in the context of a study. And secondly, clinicians in this study mainly have experience of antigen POCTs, where as reference 16 was regarding a molecular POCT.

14. Thank you for your reply to my comment “4. Page 16 line 29-30: “as they can more easily identify specific viral and bacterial infections of interest in this age group”. I wonder if removing “more easily” would make this statement clearer.

VERSION 3 - AUTHOR RESPONSE

Comments to the Author:
Thank you again for this interesting study and thank you very much for taking the time to reply to my comments and update the manuscript. My comments are below.

Abstract
1. I think the following addition at the end of the objectives sentence would help clarify what the study involved “based on their experience in routine practice”

RESPONSE: Thank you for your contribution. We have incorporated the change into the manuscript.

2. I think the conclusion may be better worded as “This study highlights how clinicians perceive respiratory tract infection tests to aid prescribe decisions amid uncertainty”

RESPONSE: Thank you for your message. We have made the change in the manuscript accordingly.

Methods
3. I think the description of the design is still somewhat confusing. E.g. “Abstract: Design. A qualitative study design was employed, using semi-structured interviews conducted in two focus group discussions.” I would suggest re-wording as: Design. A qualitative study design was employed. Two focus group discussions were conducted. I would tend to refer to the guide used for the FGDs as a topic guide rather than using the word ‘interviews’ as I think that is confusing. I would suggest updating the methods section accordingly too (i.e. don’t use the word interview – instead discuss the semi-structured topic guide)

RESPONSE: We have made this change throughout the manuscript.

4. I think there is still some confusion about the patient and public involvement – my understanding is this section is not about whether patients were recruited to the study, but whether there was any patient and public involvement e.g. input into the design of the study and interpretation of results etc.

RESPONSE: No patients were recruited for the study, and none were involved in its design or in the interpretation of the results. We have now clarified this point in the manuscript. The perspective of general practitioners and nurses was considered particularly relevant, as they are the professionals who maintain continuous and close contact with patients in daily clinical practice. For this reason, the accumulated experience of these professionals in their routine interactions with patients was taken into account, and their perspective was deemed both representative and essential to the objectives of the study. Therefore, the participation of general practitioners was prioritized over that of specialists from other fields.

5. Could the sample strategy be added to the manuscript e.g. the answer to my question about this question in the COREQ checklist (“convenience sampling strategy”)

RESPONSE: Thank you for your observation. We have added the sentence to the manuscript.

Results

6. Thank you for including the topic guide. It seems some sections of the topic guide aren't reported e.g. the information clinicians would want about a POCT to feel confident introducing it. I think this would be very useful information to report.

REVIEWER: We agree with the reviewer. We have added the following paragraph to Theme 1 in the Results section: 'Healthcare professionals required the guidelines established in clinical protocols to be clear and specific in order to make informed decisions about when it is most appropriate to use one technique over another. For this reason, there is a recognised need for more studies that provide solid evidence and concrete recommendations.'

7. I don't think this query has been answered "There seems to be a suggestion in the text the authors have written that host response POCTs (e.g. CRP) are useful for prognosis but not for diagnosis. I don't think this is always the case and host response POCTs are used diagnostically too. I think it would be useful to make it clear if that is what the authors belief (and if so perhaps provide a reference) or if that was the expressed views of the participants. If the latter some critique of this perception might be useful." I think the way that the question has been worded in the study suggests that CRP tests only provided prognostic information, not diagnostic information, which in my view isn't the case (e.g. CRP tests might be used to help distinguish viral from bacterial infection). Perhaps this could be discussed/referenced in the manuscript?

RESPONSE: We have rephrased the following paragraph in light of the suggestion:

Original:

"Regarding whether clinicians prefer techniques that provide aetiology —with microbiological tests or prognosis with CRP rapid tests when deciding whether to treat with antibiotics, participants were divided."

Revised version:

"Regarding whether clinicians prefer techniques that provide information on aetiology, such as microbiological tests, or those that support prognostic assessment, such as CRP rapid tests, which can also contribute to the diagnostic process, participants were divided in their preferences when deciding on antibiotic treatment."

8. I don't follow the answer to my comment: "There's also a suggestion running through the text that if clinicians know the patient has a bacterial infection then they know antibiotics are needed. I think it would be interesting to know if clinicians discussed this in the interviews and there were dissonant views around needing to know the prognosis rather than the diagnosis to guide antibiotic prescription?"

RESPONSE: Some professionals considered microbiological tests more important, as highlighted in the Results section, while others found CRP testing more useful because it provides information about prognosis. However, all participants agreed that both types of tests are complementary, and there was no dissonance on this point during the focus group discussions. We have clarified this in the manuscript.

9. Thank you for your reply to my comment "3. I wonder if more comparison of the different views and any dissonance between the NE and E groups might be valuable. For example, some sections of the results only have quotes from the E group – were these views held by the NE group as well?". I wonder if it would be value to report the uncertainties and hesitations that you mention of the NE group?

RESPONSE: We agree. A sentence has been added to the Results section to address this point.

10. Thank you for your reply to my comment "5. Page 10, line 41 – is there a reference for this statement? Or is it a patient perception, in which case this should be clarified". I can't see that a reference has been added to this statement in the text of the results section? ("This issue is already

occurring with CRP rapid testing in some Scandinavian countries.”)

RESPONSE: Reference included.

Discussion

11. I think a key limitation is the very little experience across both E and NE groups of using molecular tests – I think this should be reflected in the discussion, making it clear that most clinicians in the study only had experience of antigen tests.

RESPONSE: We have added this as a limitation, noting that the majority of participants in both the E and NE groups have experience using antigen tests, while their expertise with molecular tests is more limited.

12. Thank you for your reply to my comment “P14 line 42 and line 47 and P15 line 18 – reference 16 also reports these findings. Authors should note that this has already been report and that their study found similar.” I think this change has been made for p14 line 42 but not for the other findings I noted similarity with reference 16.

RESPONSE: We have removed this, and as noted in the subsequent response, we highlight the main difference with reference number 16.

13. I think it might be useful to emphasis the differences between this study and reference 16 (to emphasize the novelty of your findings). Firstly that reference 16 was conducted in the context of a trial of a rapid microbiological POCT, where as here clinicians are likely reporting ‘real life’ use of POCTs rather than in the context of a study. And secondly, clinicians in this study mainly have experience of antigen POCTs, where as reference 16 was regarding a molecular POCT.

RESPONSE: We have added: ‘Unlike the British study, which is a clinical trial focused on molecular POCTs, our study is set within real-world clinical practice and includes all types of available tests [16].’

14. Thank you for your reply to my comment “4. Page 16 line 29-30: “as they can more easily identify specific viral and bacterial infections of interest in this age group”. I wonder if removing “more easily” would make this statement clearer.

RESPONSE: Thank you for the comment. We have removed this sentence from the manuscript.

TO THE EDITOR AND REVIEWER 2. We would like to acknowledge the valuable effort made by Emily Brown as a reviewer, as her input has significantly improved the manuscript. We have not yet included your name, as we need your informed consent. However, we believe it would be appropriate and appreciated to do so. Please let us know if we have your permission to include your name.
